# Incidence and risk factors of loss to follow-up among HIV-infected children in an antiretroviral treatment program

**DOI:** 10.1371/journal.pone.0222082

**Published:** 2019-09-17

**Authors:** Suttipong Kawilapat, Nicolas Salvadori, Nicole Ngo-Giang-Huong, Luc Decker, Suparat Kanjanavanit, Achara Puangsombat, Kanchana Preedisripipat, Narong Lertpienthum, Noppadon Akarathum, Jutarat Mekmullica, Ussanee Srirompotong, Marc Lallemant, Sophie Le Coeur, Patrinee Traisathit, Charline Leroi, Gonzague Jourdain

**Affiliations:** 1 Institut de recherche pour le développement (IRD, France), U174 –PHPT, Chiang Mai, Thailand; 2 Faculty of Associated Medical Sciences, Chiang Mai University, Chiang Mai, Thailand; 3 Graduate Program in Applied Statistics, Department of Statistics, Faculty of Science, Chiang Mai University, Chiang Mai, Thailand; 4 Department of Immunology and Infectious Diseases, Harvard T.H. Chan School of Public Health, Boston, MA, United States of America; 5 Nakornping Hospital, Chiang Mai, Thailand; 6 Samutprakarn Hospital, Samutprakarn, Thailand; 7 Chiangrai Prachanukroh Hospital, Chiang Rai, Thailand; 8 Buddhachinaraj Hospital, Phitsanulok, Thailand; 9 Sanpatong Hospital, Chiang Mai, Thailand; 10 Bhumibol Adulyadej Hospital, Bangkok, Thailand; 11 Khon Kaen Hospital, Khon Kaen, Thailand; 12 Institut National d'Etudes Démographiques (INED), Paris, France; 13 Data Science Research Center, Department of Statistics, Faculty of Science, Chiang Mai University, Chiang Mai, Thailand; Katholieke Universiteit Leuven Rega Institute for Medical Research, BELGIUM

## Abstract

**Introduction:**

The success of antiretroviral treatment (ART) programs can be compromised by high rates of patient loss to follow-up (LTFU). We assessed the incidence and risk factors of LTFU in a large cohort of HIV-infected children receiving ART in Thailand.

**Methods:**

All children participating in a multicenter cohort (NCT00433030) between 1999 and 2014 were included. The date of LTFU was 9 months after the last contact date. ART interruption was defined as ART discontinuation for more than 7 days followed by resumption of treatment. Baseline and time-dependent risk factors associated with LTFU were identified using Fine and Gray competing risk regression models with death or referral to another hospital as competing events.

**Results:**

Of 873 children who were followed during a median of 8.6 years (interquartile range 4.5–10.6), 196 were LTFU, 73 died, and 195 referred. The cumulative incidence of LTFU was 2.9% at 1 year, 7.3% at 5 years and 22.2% at 10 years. Children aged 13 years and more had a 3-fold higher risk (95% confidence interval 2.06–4.78) of LTFU than those younger. Children who had interrupted ART within the previous year had a 2.5-fold higher risk (1.12–5.91) than those who had not. The risk of LTFU was lower in children stunted (height-for-age Z-scores <-2 SD) (0.42–0.96) or underweight (weight-for-age Z-scores <-2 SD) (0.24–0.97).

**Conclusion:**

Adolescence, ART interruption and absence of growth deficit were associated with LTFU. These may be warnings that should draw clinicians’ attention and possibly trigger specific interventions. Children with no significant growth retardation may also be at risk of LTFU.

## Introduction

Loss to follow-up (LTFU) among HIV-infected children on antiretroviral therapy (ART) is associated with treatment discontinuation, which in turn negatively may affect clinical outcomes and increase the risk of hospital admission or death [1–9].

Reported incidence rates (IR) of LTFU in children after ART initiation have been highly variable: from 4.2 to 18.2 per 100 child-years depending on studies [10–13]. During the first year of follow-up, rates reported in country-specific or multi-country studies varied from 4% to 19% in Africa and Asia [10, 13–16]. A cohort study of the TREAT Asia Pediatric HIV Observational Database (TApHOD) conducted in Cambodia, India, Indonesia, Malaysia, and Thailand estimated that the cumulative LTFU rate was a 24% five years after treatment initiation [10].

Several characteristics of children have been reported associated with the risk of LTFU, and results have been sometimes conflicting: immunosuppression [12, 13, 17], regimen substitution [17], diarrhea [11], wasting [13, 15], poor nutritional status [11], infancy and very young age [11, 13], but also adolescence (11–19 compared to ≤10 years) [17], mother as primary caregiver [13], calendar years [11, 13], and factors related to the organization of care [11, 15].

LTFU cases may lead to an under-estimation of mortality rates and an overestimation of a program efficiency. A meta-analysis conducted among HIV-infected children initiating ART in Southern Africa found that the mortality rate would be almost twice higher, would LTFU be taken into account [14]. We estimated the incidence and studied factors associated with LTFU in a large cohort of HIV-infected children on ART in Thailand.

## Materials and methods

### Study design and population

Data analyzed were prospectively collected between January 1, 1999 and December 31, 2014 from all HIV-infected children aged <18 years participating in the Program for HIV Prevention and Treatment (PHPT) prospective multicenter cohort study (ClinicalTrials.gov: NCT00433030) that was implemented in 43 hospitals located throughout Thailand.

### Follow-up and data collection

Children were seen by a physician or a specialized nurse at ART initiation (baseline), 2 weeks, 1, 3, 6 months and every 6 months thereafter. Demographics, clinical and laboratory data (complete blood count, chemistry and virologic data) were collected at each visit. Compensation for visit transportation cost was provided as needed. Efforts were made to trace children who were LTFU through telephone calls and home visits.

### Variables

The baseline variables assessed for association with the risk of LTFU were: sex, age, country of birth, type of hospital of birth, relationship with the caregiver, height-for-age and weight-for-age using growth standard of Thai children [18], period of ART initiation, HIV stage based on Centers for Disease Control and Prevention (CDC) classification, HIV-RNA load, CD4 percentage, and anemia status based on World Health Organization (WHO) criteria [19]. Time-dependent variables assessed were: age, height-for-age, weight-for-age, HIV CDC stage, HIV-RNA load, CD4 percentage, anemia status, ART interruptions (defined as discontinuation for more than 7 days followed by resumption), and switches of ART regimen.

### Statistical analysis

A child was considered LTFU when he/she has not attended HIV clinic for more than 9 months (3 months later than the scheduled visit) despite repeated attempts to reach him/her with telephone calls or home visits.

Continuous variables are described using medians and interquartile ranges (IQR), and categorical variables are presented as frequencies and percentages.

The cumulative incidence of LTFU was estimated using a cumulative incidence function accounting for death and referral to another hospital as competing events. The 95% confidence intervals (CIs) of estimated incidence rates were calculated based on the log-log transformation [20].

Baseline and time-dependent risk factors associated with LTFU were identified using Fine and Gray competing risk regression models [21] with death and referral to another hospital considered as competing events. All models were adjusted on the region of follow-up (northern Thailand versus others regions) to account for regional differences and on the period of ART initiation, i.e. before or after the establishment of the national AIDS treatment program in 2005 [22].

P-values from models were derived from the Wald test [23]. Missing values at baseline were imputed using the available data nearest to ART initiation (within 1 year before or, if still missing, within 15 days after), and missing values for time-dependent variables were imputed using linear interpolation [24] based on the previous and following observed values within 1 year before and after. Factors with a p-value lower than 0.25 in the univariable analysis [25] were included in the multivariable analysis, and a backward elimination procedure was used to identify factors independently associated with LTFU. Possible interactions between factors in the final model were assessed. The validity of the proportional hazards assumption for each variable was tested using time-varying interaction terms [23, 26].

All analyses and missing data imputation were performed using Stata 12.0 or R 3.4.0.

### Ethical considerations

After receiving information about the study, parents or guardians of the participating children provided written consent before enrollment. All data were collected anonymously using patient identification numbers. The PHPT cohort study protocol was approved by the ethics committees at the Thai Ministry of Public Health and local hospitals.

## Results

### Population characteristics

Of 873 children included in the analysis, 471 (54%) were female. Baseline characteristics of the population are presented in Table 1. The median age was 6.5 years (interquartile range [IQR]: 2.4–9. 6); the majority of children (61%) were followed up in the northern region of Thailand and most (91%) were living with their relatives (mother, father, grandparents, or other relatives). The median HIV-RNA load was 5.2 log_10_ copies/mL (4.7–5.7) and CD4 percentage 8% (2–16) (Table 1).

**Table 1:** Children’s characteristics at antiretroviral therapy initiation.

Characteristics	Frequency (percentage) or median [interquartile range]					
	Male (n = 402)		Female (n = 471)		All children (n = 873^a^)	
Age (years)			6.8		[2.7, 9.3]		6.5	[2.0, 10.0]	6.5	[2.4, 9.6]
Country of birth: Thailand (n = 797)			356		(98%)		425	(98%)	781	(98%)
Born in a district hospital (n = 515)			101		(45%)		151	(52%)	252	(49%)
Living with relatives (n = 730)			303		(91%)		362	(91%)	665	(91%)
Follow-up in the northern region of Thailand			236		(59%)		296	(63%)	532	(61%)
Height-for-age Z-scores (n = 602)			-2.17		[-3.06, -1.01]		-1.91	[-2.86, -1.06]	-2.03	[-3.00, -1.04]
Height-for-age Z-scores <-2 SD^b^ (n = 602)			152		(55%)		152	(47%)	304	(51%)
Weight-for-age Z-scores (n = 602)			-1.20		[0.173, -0.69]		-1.19	[-1.72, -0.59]	-1.20	[-1.73, -0.63]
Weight-for-age Z-scores <-2 SD^b^ (n = 602)			47		(17%)		52	(16%)	99	(16%)
Initiated ART before 2005			249		(62%)		302	(64%)	551	(63%)
Initiate ART before enrollment to the cohort			122		(30%)		138	(29%)	260	(30%)
CDC stage (n = 782)										
	Class N or A		162		(45%)		188	(45%)	350	(45%)
	Class B or C		198		(55%)		234	(55%)	432	(55%)
HIV-RNA load (log_10_ copies/mL) (n = 520)			5.1		[4.7, 5.6]		5.2	[4.7, 5.8]	5.2	[4.7, 5.7]
CD4 percentage (n = 650)			8		[2, 15]		9	[3, 17]	8	[2, 16]
Blood platelets (cells/mm^3^) (n = 591)			306500		[227500, 395000]		302000	[224000, 384000]	304000	[227000, 390000]
White blood cell count (cells/mm^3^) (n = 618)			7000		[5270, 9300]		7510	[5500, 10300]	7300	[5410, 10000]
Absolute neutrophil count (cells/mm^3^) (n = 585)			3080		[2180, 4218]		3325	[2409, 4981]	3276	[2270, 4633]
Absolute lymphocyte count (cells/mm^3^) (n = 616)			2380		[1550, 4120]		2550	[1584, 4670]	2465	[1571, 4359]
Anemia (WHO criteria)^c^ (n = 607)										
	No or mild anemia		129		(48%)		174	(51%)	303	(50%)
	Moderate or severe anemia		140		(52%)		164	(49%)	304	(50%)

Abbreviations: ART, Antiretroviral treatment; SD, Standard deviation; CDC, Centers for Disease Control and Prevention; WHO, World Health Organization

^a^ Except if specified otherwise

^b^ According to Thai weight and height reference values for children [18]

^c^ Anemia status according on WHO criteria adjusted on sex and age [19]

### Cumulative incidence of loss to follow-up

**Fig 1:**
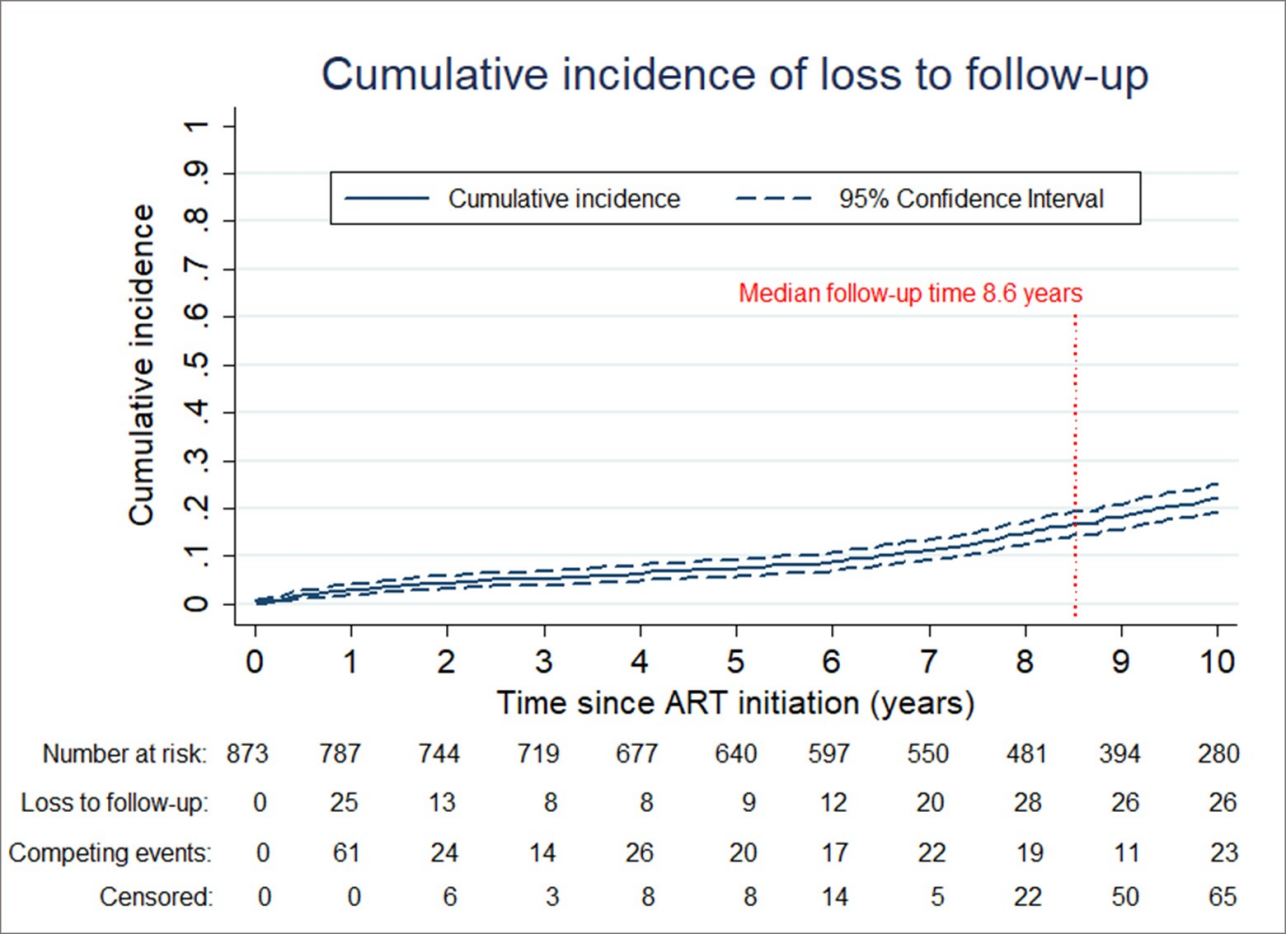
Cumulative incidence of loss to follow-up, with death and referral to another hospital accounted for as competing events.

The median duration of follow-up was 8.6 years (IQR, 4.5–10.6). During the study period, 73 children died, 196 were LTFU and 195 were referred to another hospital. A total of 38 experienced at least once ART interruption for >1 week, including 4 who died. The overall incidence rate of LTFU was 2.92 per 100 child-years (2.53–3.35). Considering death and referral to another hospital as competing events, the cumulative incidence of LTFU was 2.9% (95% CI: 1.9–4.1%) at 1 year, 7.3% (5.7–9.1%) at 5 years and 22.2% (19.3–25.2%) at 10 years (Fig 1).

### Factors associated with loss to follow-up

In the univariable analysis adjusting for the period of ART initiation and region in Thailand, baseline variables associated with a higher risk of LTFU were: age ≥7 years (subhazard ratio = 1.58; 95% CI = 1.19–2.09) and living with relatives (2.58; 1.27–5.22). Time-dependent variables associated with LTFU within the previous year were: age ≥13 years (2.81; 1.93–4.10), stunting (height-for-age Z-scores < -2 SD) (0.51; 0.34–0.77), underweight (weight-for-age Z-scores <-2 SD) (0.44; 0.25–0.77), moderate or severe anemia (0.63; 0.41–0.97), and ART interruption (2.60; 1.17–5.80) (Table 2).

**Table 2:** Association between baseline and time-dependent characteristics and loss to follow-up.

	Univariable analysis^a^			Multivariable analysis^a^ (N = 769)		
Variables	SHR	(95% CI)	*p*	aSHR	(95% CI)	*p*
**At time of ART initiation (baseline)**						
Female	1.09	(0.83–1.45)	0.53			
Age ≥7 years	1.58	(1.19–2.09)	0.002			
Country of birth other than Thailand	1.51	(0.63–3.57)	0.35			
Born in district hospital	1.10	(0.76–1.58)	0.62			
Living with relatives (versus in an orphanage)	2.58	(1.27–5.22)	0.008			** **
Height-for-age Z-scores <-2 SD^b^	0.87	(0.63–1.20)	0.40			
Weight-for-age Z-scores <-2 SD^b^	0.83	(0.52–1.34)	0.45			
ART initiation at or after enrollment to cohort	1.21	(0.88–1.67)	0.23			
CD4 ≤10%	1.03	(0.74–1.42)	0.87			
HIV RNA load >100,000 copies/mL	0.93	(0.66–1.32)	0.69			
CDC stage (class B or C)	0.81	(0.61–1.07)	0.14			** **
Moderate or severe anemia (WHO criteria)^c^	0.82	(0.59–1.14)	0.23			
**Time-dependent (within the previous year)**						
Age ≥13 years	2.81	(1.93–4.10)	<0.001	3.14	(2.06–4.78)	<0.001
Height-for-age Z-scores <-2 SD^b^	0.51	(0.34–0.77)	0.001	0.63	(0.42–0.96)	0.03
Weight-for-age Z-scores <-2 SD^b^	0.44	(0.25–0.77)	0.004	0.43	(0.24–0.77)	0.005
CD4 ≤25%	1.04	(0.75–1.45)	0.80			
HIV RNA load >400 copies/mL	1.31	(0.92–1.87)	0.13			
CDC stage (class B or C)	0.88	(0.62–1.25)	0.48			
Moderate or severe anemia (WHO criteria)^c^	0.63	(0.41–0.97)	0.03			
ART interruption^d^	2.60	(1.17–5.80)	0.02	2.57	(1.12–5.91)	0.03
At least one switch of regimen	1.34	(0.93–1.93)	0.11			** **

Abbreviations: LTFU, Loss to follow-up; n, Number of children who were LTFU in the category; N, Number of children in the category; SHR, Subhazard ratio; aSHR, Adjusted subhazard ratio; CI, Confidence intervals; ART, Antiretroviral treatment; SD, Standard deviation; CDC, Centers for Disease Control and Prevention; WHO, World Health Organization

^a^ Competing events: death, referral to another hospital; adjusting for calendar year from ART initiation and region in Thailand; missing baseline values imputed using the nearest available data within 1 year before or within 15 days after ART initiation, and missing values for time-dependent variables imputed using linear interpolation based on the previous and following observed values within 1 year

^b^ According to Thai weight and height reference values for children [18]

^c^ Anemia status based on hemoglobin level, sex, and age following WHO criteria [19]

^d^ ART interruption considered as discontinuation for more than 7 days followed by resumption

In the multivariable analysis adjusting for the period of ART initiation and region, the time-dependent variables associated with a higher risk of LTFU were age ≥13 years (adjusted subhazard ratio = 3.14;95% CI = 2.06–4.78) and ART interruption (2.57; 1.12–5.91), and the variables associated with a lower risk were stunting (0.63; 0.42–0.96), and underweight (0.43; 0.24–0.77) (Table 2). There was no significant interaction between the factors associated with the risk of LTFU (S1 Table).

## Discussion

In this cohort of 873 HIV-infected children followed for a median of 8 years in an ART program in Thailand, 196 were LTFU. At 1 year, the cumulative incidence of LTFU was 2.9%, a lower incidence than in studies using similar LTFU definition, i.e. no return to HIV clinics within the 9 months after last visit: 18.8% in a study including 11 cohorts from four countries in southern Africa [16]. The incidence seems also lower than in studies using a shorter time after last visit to define a LTFU event: 7% in a study in four southern Africa countries (6 months) [14], 7.3% in one study in South Africa (6 months) [13], and 6.9% in Nigeria (3 months) [15]. In contrast, the LTFU rate reported in Asia by TApHOD (12 months before a child is considered LTFU) [10] was only 4.0%, closer to our findings. At 5 years, the cumulative incidence rate of LTFU, 7.3%, was also lower than the 24.0% LTFU incidence reported in TApHOD [10]. Differences in LTFU rate estimates may reflect some heterogeneity between settings in term of HIV care experience, local policies and organization of care, as well as sociodemographic characteristics of children.

We considered both baseline and time-dependent variables for the evaluation of risk factors of LTFU. Interestingly, the risk of LTFU seemed more related to events arising during the follow-up than factors known at treatment initiation. We found that adolescence was a strong risk factor of LTFU, similar to a study in Ethiopia where the risk of LTFU in HIV-infected adolescents (aged 11–19 years) was 2.1 times higher than in younger children [17]. Other pediatric studies did not study the role of age at the time of loss to follow-up [11–13, 15]. A study in western Kenya reported that 16% of HIV-infected LTFU did not return to the clinic because of fear of disclosure or discrimination within the family and community [27].

In addition to adolescence, we found that transient ART interruption during follow-up was associated with a higher risk of LTFU. To our knowledge, there have been no studies assessing the association between ART interruption and risk of LTFU.

As opposed to a previous study in South Africa where underweight children at ART initiation were at a higher risk of LTFU [13], we found that stunted and/or underweight children were actually at a lower risk of LTFU. This could indicate that children who have -or have regained- a normal or suboptimal nutritional appearance may be more likely to interrupt from their hospital follow-up. Caregivers concerned with a child’s health may be reassured when he/she is no longer stunted or excessively thin and may be less motivated for regular follow-up.

In addition to the quality of the data available (prospectively collected over a median follow-up of 8.6 years and a limited number of missing data), a strength of our study was the availability of longitudinal data to evaluate the effect of changes in some key variables during the follow-up. A limitation of our study is the lack of data on some factors that other studies found associated with LTFU, such as the organization of care in each clinic [15], the distance between place of residence and clinic [28], or stigma and discrimination problems [27].

## Conclusions

Beside the increased risk of LTFU in adolescents, any ART interruption may be a warning that should trigger specific interventions and maybe prompt for enhanced communication with families and children to promote the need for close monitoring. Optimal growth should not be taken as reason for relaxing measures to ensure long term follow-up.

## Supporting information

S1 Dataset De-identified dataset used for the analysis. (XLSX)

S1 Table Assessment of potential interactions between factors associated with the risk of loss to follow-up. (DOCX)
