# Supplementary material for: Incidence and risk factors of loss to follow-up among HIV-infected children in an antiretroviral treatment program
Source: PLoS One. 2019 Sep 17;14(9):e0222082. doi: 10.1371/journal.pone.0222082 (PMC6748564; doi:10.1371/journal.pone.0222082)
Supplement: S1 Table — (DOCX) [file pone.0222082.s002.docx]

**S1 Table. Assessment of potential interactions between factors associated with the risk of loss to follow-up**

| **Variables** | ***p*** | **Conclusion^a^** |
| --- | --- | --- |
| **Age x Height-for-age Z-scores**^b^ |  | No interaction |
| Age | <0.001 |  |
| Height-for-age Z-scores | 0.212 |  |
| Interaction | 0.194 |  |
| **Age x Weight-for-age Z-scores**^b^ |  | No interaction |
| Age | <0.001 |  |
| Weight-for-age Z-scores | 0.010 |  |
| Interaction | 0.467 |  |
| **Age x ART interruption**^c^ |  | No interaction |
| Age | <0.001 |  |
| ART interruption | 0.229 |  |
| Interaction | 0.465 |  |
| **Height-for-age Z-scores x Weight-for-age Z-scores** |  | No interaction |
| Height-for-age Z-scores | 0.034 |  |
| Weight-for-age Z-scores | 0.091 |  |
| Interaction | 0.446 |  |
| **Height-for-age Z-scores x ART interruption** |  | No interaction |
| Height-for-age Z-scores | 0.003 |  |
| ART interruption | 0.572 |  |
| Interaction | 0.036 |  |
| **Weight-for-age Z-scores x ART interruption** |  | No interaction |
| Weight-for-age Z-scores | 0.008 |  |
| ART interruption | 0.056 |  |
| Interaction | 0.634 |  |

Abbreviations: ART, Antiretroviral treatment

^a^ Interaction was considered significant when p-values of all terms were significant

^b^ According to Thai weight and height reference values for children

^c^ ART interruption: discontinuation for more than 7 days followed by resumption
